# Predicting offer burden to optimize batch sizes in simultaneously expiring kidney offers

**DOI:** 10.3389/frai.2025.1662960

**Published:** 2025-09-18

**Authors:** Sean Berry, Berk Görgülü, Sait Tunç, Mucahit Cevik

**Affiliations:** ^1^Department of Mechanical, Industrial and Mechatronics Engineering, Toronto Metropolitan University, Toronto, ON, Canada; ^2^DeGroote School of Business, McMaster University, Hamilton, ON, Canada; ^3^Grado Department of Industrial and Systems Engineering, Virginia Polytechnic Institute and State University, Blacksburg, VA, United States

**Keywords:** organ nonuse, simultaneously expiring offers, survival models, decision support, machine learning, AI interpretability

## Abstract

**Background:**

Timely and efficient allocation of deceased donor kidneys is a persistent challenge in transplantation. Traditional sequential offer systems often lead to extended delays and high nonuse rates, as many kidneys undergo multiple refusals before being accepted. Simultaneously expiring offers, where a kidney is offered to a batch of centers with synchronized response deadlines, offer a more efficient alternative. However, fixed batch sizes fail to account for variability in offer requirements, potentially introducing new inefficiencies or overwhelming transplant professionals with excessive notifications.

**Methods:**

We investigated the use of machine learning-based survival models to dynamically predict the number of offers a kidney will require before acceptance. Utilizing data on over 16,000 deceased donor kidneys from the national organ offer dataset, we engineered predictive features from both donor profiles and recipient pool characteristics. We trained and evaluated multiple survival models using time-dependent concordance indices along with other survival and regression performance metrics.

**Results:**

The Random Survival Forest model achieved the best performance, with a time-dependent C-index of 0.882, effectively estimating the required offer volume for kidney placement. Feature importance analysis revealed that waitlist characteristics, such as mean Estimated Post-Transplant Survival (EPTS), mean Calculated Panel Reactive Antibody (CPRA), time on dialysis, and waitlist duration, were among the most influential predictors. When integrated into a dynamic simultaneous offer system, these predictions have the potential to reduce average placement delays from 17.37 h to 1.59 h while maintaining a manageable level of extraneous offers.

**Discussion:**

Our results demonstrate that survival-based predictive modeling can meaningfully improve the efficiency of simultaneously expiring offers in kidney allocation. By personalizing batch sizes based on expected offer burden, such models can reduce delays without overwhelming transplant professionals. These findings underscore the value of integrating real-time, data-driven tools into organ allocation systems to improve operational efficiency and facilitate practical implementation.

## 1 Introduction

Kidney transplantation is the preferred treatment for end-stage kidney disease, offering superior survival and quality-of-life outcomes compared to dialysis (Wolfe et al., 1999), along with substantial long-term cost savings (Laupacis et al., 1996). Yet despite the growing need, kidney nonuse remains a major challenge. In 2023, the nonuse rate reached a record high of 27.9 (Israni et al., 2025). The allocation of deceased donor kidneys is a complex, time-sensitive process involving real-time decisions by transplant centers, shaped by donor characteristics, recipient compatibility, and institutional constraints (Zhang et al., 2023). As a result, many kidneys undergo extended sequences of refusals: one-quarter of transplanted kidneys are first offered to at least 73 candidates. These delays increase Cold Ischemia Time (CIT)—the duration an organ remains preserved before transplantation—which is associated with reduced graft function and survival (Lum et al., 2023). Prolonged offer sequences also create significant logistical burdens. Some transplant centers receive up to 700 offers per month (Reddy et al., 2022), contributing to operational fatigue and resource strain. Ultimately, this inefficiency delays patient access to transplantation and increases the risk of graft failure or kidney nonuse.

Despite ongoing policy reforms, the kidney allocation system continues to face significant challenges in improving organ utilization (Israni et al., 2025). Most recently, the 2021 implementation of KAS250 replaced regional boundaries with a 250-nautical-mile radius allocation framework, aiming to reduce geographic disparities. However, this change has introduced new logistical burdens and operational inefficiencies for transplant centers (Yu et al., 2025). Although there is broad recognition among patients and clinicians of the need to reduce kidney nonuse, support for more risk-tolerant allocation policies remains divided (Mehrotra et al., 2020). National efforts to expand the donor pool—such as increased use of donation after circulatory death and the adoption of hypothermic machine perfusion to extend acceptable CIT—have not yielded the expected improvements in utilization (McKenney et al., 2024). Compounding these issues is the substantial variation in acceptance behavior, not only across transplant centers but also among clinicians within the same center, even after adjusting for organ quality (Green et al., 2025). This inconsistency further limits the effectiveness of policy-level interventions and underscores the need for more adaptive, data-driven approaches to kidney allocation.

Simultaneously expiring offers have been proposed as a strategy to reduce kidney nonuse and accelerate organ placement. Under this system, a kidney is offered to multiple transplant centers simultaneously, with each center required to respond within a fixed time window. This contrasts with traditional sequential offers and allows more offers to be made within a shorter period (Mankowski et al., 2019). Simulation studies suggest that this approach can improve organ utilization and reduce delays. However, current implementations typically apply a fixed batch size across all offers, regardless of donor quality or anticipated placement difficulty. This uniform approach can lead to inefficiencies: high-quality kidneys may be over-offered, while marginal kidneys may still face long placement times. Additionally, offering organs too broadly increases the likelihood that centers invest time evaluating an offer only to be bypassed—contributing to decision fatigue and diminishing willingness to fully engage with future offers (Carminati, 2020), considering that a center accepting an organ offer can be ultimately bypassed due to a higher-priority acceptance on the match run.

Clinical decision-making in organ transplantation imposes a substantial cognitive load on providers, who must quickly interpret complex and uncertain information under time pressure. In kidney transplantation, offers often arrive unpredictably and in bursts, requiring repeated assessments of donor quality, recipient compatibility, and logistical feasibility. This high volume of time-sensitive decisions contributes to *decision fatigue*—the gradual decline in decision quality resulting from sustained mental effort (Pignatiello et al., 2020). The adoption of simultaneously expiring offers, while aimed at improving allocation efficiency, has been shown to further increase cognitive demands on clinicians (Mankowski et al., 2019; Erazo et al., 2022). The consequences of decision fatigue are well-documented in clinical settings and include susceptibility to cognitive biases, reduced persistence, impulsivity, and avoidant behavior (Grignoli et al., 2025). In transplantation, these effects may manifest as delayed or suboptimal responses to offers, prolonged wait times, and missed opportunities for organ placement—outcomes with serious clinical and operational costs.

Recent work by Erazo et al. (2022) introduced a simulation-optimization framework to determine optimal batch sizes for organ offers, accounting for organ quality and location. Their policy assigns batch sizes based on predefined organ categories (e.g., KDRI ranges for kidneys) and Organ Procurement Organization (OPO) location, using simulation outcomes to maximize system-wide utility. While this approach demonstrates clear improvements in organ utilization and time-to-allocation over fixed batch-size policies, it relies heavily on extensive simulation infrastructure and does not produce organ-level predictions. Instead, it applies group-level policies determined offline, without incorporating real-time donor- or match-run-specific context. As a result, while effective in aggregate, these strategies may miss opportunities for more precise, data-driven tailoring of batch sizes that reflect individual offer complexity or waitlist dynamics.

The growing availability of rich, high-dimensional clinical data has enabled the use of machine learning to improve predictive modeling in healthcare (Ravindhran et al., 2023). Recent studies in transplantation have leveraged machine learning to address diverse prediction tasks, such as pre-transplant mortality, organ nonuse, graft survival, post-transplant complications, and long-term patient outcomes (Massie et al., 2010; Marrero et al., 2017; Li et al., 2024; Gotlieb et al., 2022; Connor et al., 2021; Berry et al., 2024; Ge et al., 2023). However, most existing work focuses on post-transplant predictions and does not address logistical challenges during the allocation process. In contrast, our study introduces a novel application of machine learning to pre-transplant logistics: predicting the number of offers a specific kidney will require before acceptance. To our knowledge, this is the first effort to develop organ-level predictions of offer burden as a mechanism to guide dynamic batch sizing in simultaneously expiring offers. By moving beyond fixed or category-based batching strategies, our approach enables real-time, individualized offer design based on donor, waitlist, and match-run context.

In addition to predictive accuracy, clinical deployment of such models requires interpretability. As machine learning models grow in complexity, transparency in decision-making is essential to build trust among clinicians. While traditional models like the Cox Proportional Hazards model offer intrinsic interpretability, recent efforts have increasingly adopted more flexible models combined with *post-hoc* explanation techniques, such as SHAP (SHapley Additive exPlanations) (Berchuck et al., 2024; Gogoi and Valan, 2024). Our framework leverages this paradigm to provide both predictive power and actionable insights into the drivers of offer burden.

The aim of this study is to leverage survival-based machine learning models to predict how many offers a deceased donor kidney will require before acceptance, and to show how these predictions can inform dynamic, organ-specific batch sizing for more efficient allocation. We note that the individualized predictions by the survival models offer a flexible alternative to static, fixed-size offer groups in simultaneously expiring offers. Using comprehensive national transplant data, we simulate multiple allocation strategies, including the current sequential match run and a range of fixed-size batch policies, and compare them in terms of offer delays and the number of extraneous offers made after an acceptance. Our results demonstrate that dynamic, prediction-informed batching can significantly reduce placement times while limiting unnecessary workload on transplant centers. To enhance interpretability and support clinical adoption, we use SHAP analysis to identify key features influencing model predictions, providing insight into the factors that drive variation in acceptance timelines.

## 2 Methodology

In this section, we first describe the dataset and feature construction process, then introduce our modeling framework and survival prediction setup. We next present the evaluation metrics used to assess model performance, followed by a simulation-based evaluation of batch policy alternatives. Finally, we discuss the interpretability methods used to explain model predictions.

### 2.1 Data description

We construct a unique dataset composed of the deceased donor data and the Potential Transplant Recipient dataset from Organ Procurement and Transplantation Network (OPTN) which documents all kidney offers to patients on the U.S. waiting list. The feature set was informed by prior research and domain expertise, aiming to balance donor-specific characteristics with aggregated proposed recipient features from the OPTN dataset to capture both organ quality and waitlist context. Donors missing key features which are used in the analysis are excluded. The dataset is composed of both key donor features, as well as summary features created through analysis of the potential recipients to whom the kidney was offered. These aggregate features approximate properties of the local match run or offer pool and serve as a proxy for the waitlist.

For the purposes of our experiment we have taken a subset of the proposed transplant recipient dataset consisting of 20 million offers made up of 16,408 distinct donors, covering the period from February, 2018 to August, 2019, thus excluding the COVID-19 period to avoid potential anomalies. After extracting the data chunk from the match run data, the corresponding offers are aggregated to just one observation per donor kidney; these aggregate feature are mostly composed of the mean, min, and max values of the waitlist. Offers without a corresponding entry to the deceased donor file were also removed, as we could not make use of several features contained therein. In addition to standard donor characteristics and waitlist-derived statistics, we incorporated several engineered features to enrich the predictive modeling process. These include candidates time on the waitlist and their distance to the transplant center, as well as two measures of center-specific acceptance patterns. The first, the age-count heuristic, counts kidneys of similar quality accepted for recipients of a similar age at the same center in the previous 2 years. The second, the 2-year greater-creatinine heuristic, counts kidneys with higher serum creatinine accepted at the same center during that period.

The complete set of continuous and categorical features used in this analysis are listed in Tables 1, 2, respectively. For the continuous features, we include detailed statistics including min, max, mean and median values to provide a clear picture of data distribution. Prior to model training, all categorical features were one-hot encoded, that is, converted into separate binary columns for each category and all continuous features were standardized. For categorical variables, all levels shown in Table 2 were retained as they appear in the OPTN source data including “Unknown” values. Furthermore, one-hot encoding these features ensures interpretability and fair comparison, as some models are less resilient to alternative encodings of categorical variables. Of note is the high variability of some of the features. For instance, CIT ranges from 0 indicating an offer was made before clamp time all the way up to rare extreme values exceeding 34,000 minutes, which are clinically implausible and likely reflect data entry anomalies. However, because we use the minimum CIT across all offers for a donor in the model, such extreme values are exceedingly rare and were retained to preserve fidelity to the original registry data and reflect real-world data conditions, under which this decision-support tool would operate. Similarly the prevalence of 0's in the min value column have clear interpretations within the dataset: donor age 0 corresponds to a small number of neonatal donors, distance 0 reflects scenarios where donor and recipient were located in the same center, and waitlist time 0 indicates candidates who received an offer on the same day they were listed, often in urgent cases. Several features in our dataset are calculated in real time for each individual offer event, prior to aggregation at the donor level, including CIT, donor-recipient distance, candidate waitlist duration, time on dialysis, and a center-specific heuristic that captures the number of kidneys with higher serum creatinine accepted over the preceding 2 years (i.e., 2-Year Greater Creatinine). These required extensive preprocessing, such as aligning donor and candidate timelines, computing offer-specific values (e.g., cold ischemia from cross-clamp time), and handling inconsistencies across sources. Several other features such as waitlist time, dialysis duration and CPRA display were heavily skewed, reflecting wide differences across patients, and further support the use of feature standardization. Among the categorical features substance use indicators, such as history of IV drug use or cocaine use, are non-negligible, highlighting the inclusion of extended criteria donors. Most clinical variables contain low levels of missing data (typically < 2%), coded as “Unknown” and retained as distinct levels.

**Table 1:** Summary statistics for continuous features.

**Feature**	**Mean (SD)**	**Min**	**25%**	**Median**	**75%**	**Max**
Min cold ischemia (Minutes)	28.76 (448.38)	0.000	0.000	0.000	0.000	34,851.000
Min KDRI	1.37 (0.49)	0.604	0.996	1.268	1.641	4.837
Min age count heuristic	216.99 (503.56)	0.000	4.564	41.274	191.387	6,385.519
Min distance (KM)	8.18 (19.98)	0.000	0.000	1.000	7.000	507.000
Min time waitlisted (Days)	314.35 (637.08)	0.000	3.000	32.000	277.250	8,875.000
Min initial EPTS	10.24 (21.16)	0.000	0.000	0.000	8.000	100.000
Min years on dialysis	1.899 (3.099)	0.000	0.000	0.000	3.105	33.791
Min recipient age (Years)	32.53 (18.20)	0.334	19.567	30.576	46.480	88.977
Min CPRA	3.577 (16.641)	0.000	0.000	0.000	0.000	100.000
Min 2-year greater creatinine	0.07 (0.41)	0.000	0.000	0.000	0.000	15.000
Mean age count heuristic	569.50 (770.83)	0.000	53.711	226.186	814.552	7,260.661
Mean distance (KM)	18.63 (24.68)	0.000	3.562	10.446	24.500	507.000
Mean time waitlisted (Days)	881.44 (650.74)	0.000	470.333	748.000	1,170.557	8,875.000
Mean initial EPTS	32.71 (20.70)	0.000	16.600	35.344	45.001	100.000
Mean years on dialysis	4.285 (2.838)	0.026	2.287	3.443	5.676	33.791
Mean recipient age (Years)	51.40 (12.23)	1.071	48.108	54.617	58.960	88.977
Mean CPRA	6.835 (17.767)	0.000	0.000	0.489	2.750	100.000
Mean 2-year greater creatinine	0.16 (0.48)	0.000	0.000	0.000	0.046	15.000
Max age count heuristic	1,319.05 (1,702.88)	0.000	92.256	429.516	2,345.306	9,476.486
Max distance (KM)	47.98 (68.77)	0.000	8.000	23.000	58.000	521.000
Max time waitlisted (Days)	2,223.30 (1,582.93)	0.000	940.000	2,101.000	3,212.000	11,790.000
Max initial EPTS	65.57 (36.87)	0.000	31.000	83.000	98.000	100.000
Max years on dialysis	9.746 (6.786)	0.000	4.884	8.631	13.303	42.122
Max recipient age (Years)	64.97 (17.50)	1.071	55.090	68.981	78.418	91.883
Max CPRA	75.582 (29.106)	1.000	60.000	89.000	99.000	100.000
Max 2-year greater creatinine	0.670 (1.283)	0.000	0.000	0.000	1.000	16.000
Donor age (Years)	40.20 (16.83)	0.000	28.000	41.000	54.000	87.000
Donor creatinine	1.45 (1.41)	0.020	0.700	1.000	1.590	35.000

CIT, Cold Ischemia Time; KDRI, Kidney Donor Risk Index; EPTS, Estimated Post-Transplant Survival; CPRA, Calculated Panel Reactive Antibody.

**Table 2:** Summary statistics for categorical features.

**Feature**	**Level**	**Mean**
Hypertension history	No	0.660
	Unknown	0.010
	Yes	0.330
Cancer history	No	0.959
	Unknown	0.009
	Yes	0.032
Cigarette use	No	0.768
	Unknown	0.021
	Yes	0.211
DCD status	No	0.928
	Yes	0.072
MI history	No	0.946
	Unknown	0.012
	Yes	0.041
Cocaine use	No	0.747
	Unknown	0.019
	Yes	0.234
IV drug use	No	0.854
	Unknown	0.016
	Yes	0.130
Donor ethnicity	White	0.670
	Black	0.148
	Hispanic	0.139
	Asian	0.024
	American Indian/Alaska Native	0.005
	Native Hawaiian/Pacific Islander	0.003
	Multiracial	0.010
Donor gender	Female	0.392
	Male	0.608
Donor blood type	Category A	0.161
	Category A1	0.169
	Category A1B	0.011
	Category A2	0.036
	Category A2B	0.005
	Category AB	0.018
	Category B	0.122
	Category O	0.477

DCD, Donation after Circulatory Death.

To give a clearer picture of the study population, we report some key demographic and clinical characteristics for the recipients. The mean recipient age was 55.73 years with a standard deviation of 12.33 years. Table 3 summarizes the distributions of blood type, race/ethnicity, gender, and primary diagnosis. The most common blood type was O (66.60%), followed by A (24.67%). The largest racial/ethnic groups were White (35.22%), Black (30.80%), and Hispanic (23.38%). The most frequent primary diagnoses were Type II diabetes mellitus (36.88%) and hypertensive nephrosclerosis (21.01%). These distributions are broadly in line with what might be expected in a kidney transplant recipient population.

**Table 3:** Distributions of blood type, race/ethnicity, gender, and primary diagnosis for recipients.

**Feature**	**Level**	**Mean**
**Blood type**		
	O	0.666
	A	0.247
	B	0.061
	AB	0.021
	A1	0.004
	A2	0.001
	A1B	0.000
	A2B	0.000
**Race/Ethnicity**		
	White	0.352
	Black	0.308
	Hispanic	0.234
	Asian	0.087
	American Indian/Alaska Native	0.007
	Native Hawaiian/Other Pac. Islander	0.004
	Multiracial	0.008
**Gender**		
	Male	0.681
	Female	0.319
**Primary diagnosis**		
	Diabetes mellitus—type II	0.369
	Hypertensive nephrosclerosis	0.210
	Polycystic kidneys	0.070
	Other specify / unknown	0.069
	Focal glomerular sclerosis (FSGS)	0.052
	IgA nephropathy	0.040
	Diabetes mellitus—type I	0.028
	Retransplant / graft failure	0.028
	Systemic lupus erythematosus	0.023
	Chronic glomerulonephritis, unspecified	0.015
	*Other diagnoses (each < 1%)*	0.097

### 2.2 Modeling approach

The organ offer process is inherently time-dependent, offers are sent to centers sequentially until one is accepted or the organ is removed from match run and not used. Crucially, the quality of the organ deteriorates over time due to increasing CIT, which is known to negatively impact transplant outcomes. As such, allocation is effectively a race against time, where delays reduce both the likelihood of acceptance and post-transplant viability. Traditional classification or regression approaches fail to capture the uncertainty involved in this process. We adopt survival modeling techniques well suited to modeling time-to-event outcomes, where event is defined as the acceptance of the organ. This allows us to leverage censored data and produce risk scores estimating the acceptance over time.

Unlike traditional classification or regression approaches, survival models are well suited for modeling time-to-event data outcomes with censoring. In our setting where observations are made up of donor kidneys and the aim is to predict offer burden, censoring occurs when the kidney is removed from the offer pool without acceptance. Crucially, these censored observations account for ~ 25% of all donor kidneys in the dataset. Ignoring them would exclude a substantial portion of the allocation process and systematically bias predictions toward cases with faster placement. Traditional regression models (e.g., OLS, random forests, or gradient boosting) can be trained on observed offer counts but typically ignore censored data and treat the target as fully observed, which may bias predictions for non-utilized organs. Survival models provide a natural framework for this task by jointly modeling the probability and timing of events while incorporating censored observations. Their established utility in kidney graft survival analysis further supports their application in modeling offer dynamics (Riley et al., 2022).

Table 4 illustrates a small survival dataset representing how many offers were made before each kidney was either accepted or labeled as non-utilized. Kidneys A, C, and D were accepted after 2, 1, and 3 offers respectively, while kidneys B and E were never accepted and are thus right-censored. Survival models leverage this structure by treating censored examples as partial observations, i.e., the kidney remained unaccepted up to at least the reported number of offers. In contrast, a standard regression or classification model cannot properly handle censored observations. It would typically need to either discard these examples entirely or assign them a fixed label, treating them as if the event did occur (at the last observed time).

**Table 4:** Illustrative example survival data in the context of kidney offers.

**Kidney ID**	**Offers made**	**Accepted**	**Censored**
A	2	Yes	No
B	5	No	**Yes**
C	1	Yes	No
D	3	Yes	No
E	4	No	**Yes**

Survival models can be further separated into classical survival models such as Cox Proportional Hazards (CPH) or Weibull Accelerated Failure Time and machine learning-based approaches. The classical approaches assume parametric or semi-parametric forms for the hazard or survival function and rely on strong assumptions such as proportional hazards or specific distributional forms. These models perform well in certain settings but may be too rigid to capture complex interactions or non-linear effects in the allocation data. Machine learning-based survival models, such as DeepSurv (Katzman et al., 2018) and Random Survival Forests (RSF) (Pölsterl, 2020), offer a hybrid approach: they handle censoring like classical models but allow for greater flexibility in learning nonlinear patterns and feature interactions (Garcia-Lopez et al., 2025; Kang, 2024). For example, the assumption of log-linearity and proportional hazards means that the effect of a high CPRA score is assumed constant over time, whereas a RSF or DeepSurv model, by contrast, can learn that CPRA interacts with factors like EPTS or CIT to change acceptance risk dynamically.

The models considered in our empirical analysis include CPH, Weibull, RSF, and DeepSurv. Although we initially evaluated DeepHit, it was ultimately excluded due to consistently lower performance on time-dependent concordance metrics relative to the other models. A key limitation of DeepHit is its reliance on discretizing the event timeline into a fixed number of intervals, which reduces the time dependent resolution of the predicted survival curves and may hinder its ability to capture nuanced event dynamics. For all remaining models, time-to-event predictions are computed consistently using the median survival time, defined as the time point at which the predicted survival probability falls below 0.5.

### 2.3 Evaluation metrics for survival models

To evaluate model performance in predicting time to kidney acceptance, we employed the time-dependent concordance index (C-index), a metric designed for survival analysis settings where event times may be censored. Unlike the standard concordance index, which assesses a model's ability to rank survival outcomes across the entire dataset, the time-dependent version evaluates the model's ranking ability at each observed event time. This is particularly well-suited to our application, where the timing of organ acceptance, not just whether acceptance occurs, is of critical importance for operational decisions and policy development.

The time-dependent concordance index is defined as:


(1) $$\begin{array}{l} \text{C-index}=\frac{\sum_{i=1}^{n}\delta_{i}\sum_{j\in R\left(T_{i}\right)}1\left(\hat{S}\left(T_{i}|x_{i}\right)<\hat{S}\left(T_{i}|x_{j}\right)\right)}{\sum_{i=1}^{n}\delta_{i}\cdot |R\left(T_{i}\right)|} \end{array}$$

where *n* denotes the total number of donor kidneys, and δ_*i*_ is an event indicator that equals 1 if kidney *i* was accepted (i.e., the event occurred) and 0 if it was censored (i.e., removed from consideration without being accepted). *T*_*i*_ is the acceptance time for kidney *i*, and $\hat{S}\left(T_{i}|x\right)$ represents the predicted survival probability at time *T*_*i*_ given the feature vector *x*. The set $R\left(T_{i}\right)$ consists of kidneys that are still at risk (i.e., not yet accepted or censored) at time *T*_*i*_, and the indicator function **1**(·) evaluates to 1 if the model correctly ranks kidney *i* as more likely to be accepted earlier than kidney *j*, based on survival probabilities at that time.

This metric captures the proportion of correctly ordered pairs among all comparable donor kidneys, averaged over all actual acceptance times. By incorporating censoring and focusing on pairwise comparisons at each event time, C-index offers a time sensitive and policy-relevant measure of predictive performance. This is particularly valuable in the context of organ allocation, where reducing CIT and improving the timing of placements are as critical as the acceptance itself. Accurately ranking which kidneys are likely to be accepted sooner allows for better offer planning and more efficient use of the donor pool. Thus, C-index provides a robust and context-appropriate metric for evaluating survival-based models in this domain.

In addition to C-index, we evaluate model performance using the Integrated Brier Score (IBS) and Negative Binomial Log-Likelihood (NBLL). IBS measures the mean squared error between predicted survival probabilities and observed acceptance outcomes across time whereas NBLL evaluates how well the model predicts the number of offers before acceptance by comparing the predicted and observed distributions. Several regression metrics are considered as well, including Mean Absolute Error (MAE) which reflects the average magnitude of the difference in offers seen vs. offers predicted, and Mean Absolute Percentage Error (MAPE) which expresses MAE as a relative percentage of the observed values. We also report Root Mean Squared Error (RMSE), and Normalized RMSE (NRMSE) which measure difference between actual and predicted offer burden while more heavily penalizing larger deviations NRMSE scales this by the range of observed values for comparability. Finally we report the coefficient of determination (*R*^2^) which indicates the proportion of variation in observed offer burden explained by the model. These metrics together capture not only the models ability to rank risks accurately but also the models ability to make accurate time to event predictions.

For each model we report the mean and standard deviation of performance across the ten evaluation folds. Model performance is also assessed using 5 × 2 repeated cross-validation. Specifically, to assess whether observed performance differences are statistically meaningful, we conduct paired *t*-tests comparing one model against another across the 5 × 2 cross-validation folds.

### 2.4 Batch policy evaluation

To evaluate the potential impact of alternative batching strategies, we provide a counterfactual analysis under alternative batch designs. In the observed match run, kidneys are offered sequentially, one candidate at a time, until an acceptance or the organ is deemed unusable. We investigate two alternative batching modes: static batch sizes where a fixed number of candidates are offered simultaneously regardless of organ or potential recipient characteristics, and dynamic batch sizes, in which the number of concurrent offers is set based on the survival model's predicted offer burden for each organ. These batch sizes are derived from the predicted median survival time or quantile-based adjustments on the predicted survival curve.

In all simulations we apply a simplified set of assumptions namely each batch is assumed to be sent at the same timestamp with a fixed 1-h expiration period, after which a new batch is issued if acceptance has not occurred. A batch is considered successful if any candidate within would have accepted the offer using the original match run as the ground truth. We then evaluate the outcomes of the policy by computing two metrics per organ, the total time elapsed until acceptance and the number of extraneous offers defined as offers made after the ground truth acceptance point. The first reflects potential delays in the placement process, which could increase CIT or risk nonuse. The second captures the additional workload imposed on centers by offering to candidates who, in practice, would not have received an offer under sequential offering. The ground truth acceptance time per organ is not assumed, but directly observed from the data, reflecting real-world offer sequences. Under this baseline extraneous offers are zero by definition, since organs are accepted in strict sequential order.

These metrics allow us to compare both the potential average decrease in wait time as well as the potential average cost in terms of offers that would not have been sent under the original match run. In particular, we are interested in whether using model-informed dynamic batch sizes can speed up organ placement while avoiding a large increase in unnecessary offers. The approach of tailoring batch sizes to each individual organ may reduce delay with less additional workload compared to static batch sizing.

### 2.5 Interpretability methods

To support interpretability in our analysis, we restricted the explanatory feature set to a curated subset of variables. Although the RSF model is capable of handling a large number of input features without overfitting, a property that makes it attractive for complex prediction tasks, this flexibility can come at the cost of interpretability. To address this, we also experimented with a reduced feature set based on domain expertise, prioritizing variables that contribute meaningfully to predictive performance while also providing clinically relevant insights.

A limitation of RSF in this context is its lack of built-in feature importance metrics or native support for *post-hoc* interpretation techniques commonly used in tree-based models. Prior studies, such as in graft failure prediction, have used permutation-based importance measures to assess relative feature relevance (Einecke et al., 2021). However, due to the limited robustness and granularity of such methods, we instead employed SHAP, a model-agnostic framework that provides local explanations grounded in cooperative game theory. SHAP assigns each feature a contribution value for a specific prediction, ensuring consistency and local accuracy in how feature effects are attributed. More specifically, we used Kernel SHAP (Lundberg and Lee, 2017), a general-purpose, model-agnostic variant well-suited for non-differentiable models like RSF. To tailor SHAP to our prediction goal, we defined the model output as the predicted median survival time, ensuring that SHAP values reflected the influence of input features on the final time-to-event prediction.

Our interpretability analysis includes both local and global SHAP explanations. Local explanations provide insight into feature contributions for individual donor cases, which is critical for our proposed application of dynamic, donor-specific batch sizing, while global explanations reveal the average impact of each feature across the dataset. Together, these perspectives allow us to understand which features extend or reduce expected time to acceptance in specific cases, and which variables drive model behavior overall.

## 3 Numerical results

This section presents the results of our experimental evaluation across three core areas. First, we assess survival model performance using standard survival and regression metrics to identify the best performing model. Next, we investigate the clinical utility of the proposed dynamic batching framework, highlighting its potential to reduce allocation delay while minimizing extraneous offers. Finally, we explore model interpretability using SHAP-based explanations to illustrate how different features influence predicted offer burden.

### 3.1 Comparative performance analysis

A comparative analysis of all model performances across survival and error-based metrics is presented in Table 5. The RSF model outperformed all others, achieving the highest concordance index (C-index = 0.869) and the lowest prediction errors across multiple metrics, including MAE (0.410), MAPE (15.14), RMSE (0.326), and NRMSE (0.041). RSF also achieved the highest coefficient of determination (*R*^2^ = 0.923), indicating strong agreement between predicted and observed time-to-acceptance. Statistical comparisons confirm RSF's superior performance across nearly all metrics. With respect to pairwise statistical comparisons between RSF and each other model, all differences were statistically significant except for three cases: RSF vs. CPH on IBS (*p* = 0.058), RSF vs. DeepSurv on NBLL (*p* = 0.137), and RSF vs. Weibull on NBLL (*p* = 0.142). In addition, CPH achieved a significantly lower NBLL than RSF (*p* < 0.001). Despite this, RSF consistently demonstrated the strongest overall performance across the majority of metrics and model comparisons.

**Table 5:** Comparison of model performance across eight evaluation metrics.

**Model**	**C-index**	**IBS**	**NBLL**	**MAE**	**MAPE**	**NRMSE**	**R^2^**	**RMSE**
**CPH**	0.857 (0.005)	**0.039** (0.002)	**0.127** (0.006)	0.468 (0.021)	18.856 (0.769)	0.051 (0.005)	0.903 (0.009)	0.407 (0.041)
**DeepSurv**	0.835 (0.018)	0.048 (0.008)	0.154 (0.025)	0.563 (0.099)	22.660 (4.258)	0.079 (0.033)	0.850 (0.066)	0.628 (0.272)
**RSF**	**0.869** (0.004)	0.040 (0.002)	0.141 (0.006)	**0.410** (0.015)	**15.137** (0.701)	**0.041** (0.003)	**0.923** (0.004)	**0.326** (0.019)
**Weibull**	0.851 (0.009)	0.046 (0.003)	0.159 (0.032)	0.531 (0.029)	22.238 (2.020)	0.096 (0.016)	0.861 (0.051)	0.755 (0.113)

Reported values are the mean and standard deviation (in parentheses) over 10-fold cross-validation. Bolded values indicate the best-performing model for each metric.

**Figure 1:**
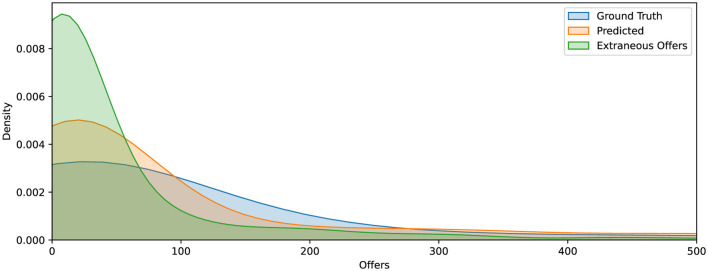
Kernel density estimates of offer burden for ground truth (blue), RSF model predictions (orange), and extraneous offers (green). The close alignment between predicted and observed distributions indicates strong model calibration. The distribution of extraneous offers is sharply skewed toward the left, suggesting that excess offers tend to cluster soon after the predicted acceptance point.

To further evaluate model performance, we plot kernel density estimates of the RSF model's predicted, observed, and extraneous offers in Figure 1. This visualization serves two purposes. First, it illustrates the alignment between predicted and actual offer burdens. The close overlap between the predicted distribution (orange) and the observed distribution (blue) indicates strong agreement across most of the range, demonstrating the model's ability to accurately estimate offer burden. Second, the sharp peak near zero in the extraneous offers distribution highlights the model's conservative batching behavior, favoring smaller batch sizes that minimize unnecessary offers while still achieving timely placements.

### 3.2 Evaluating clinical utility

**Figure 2:**
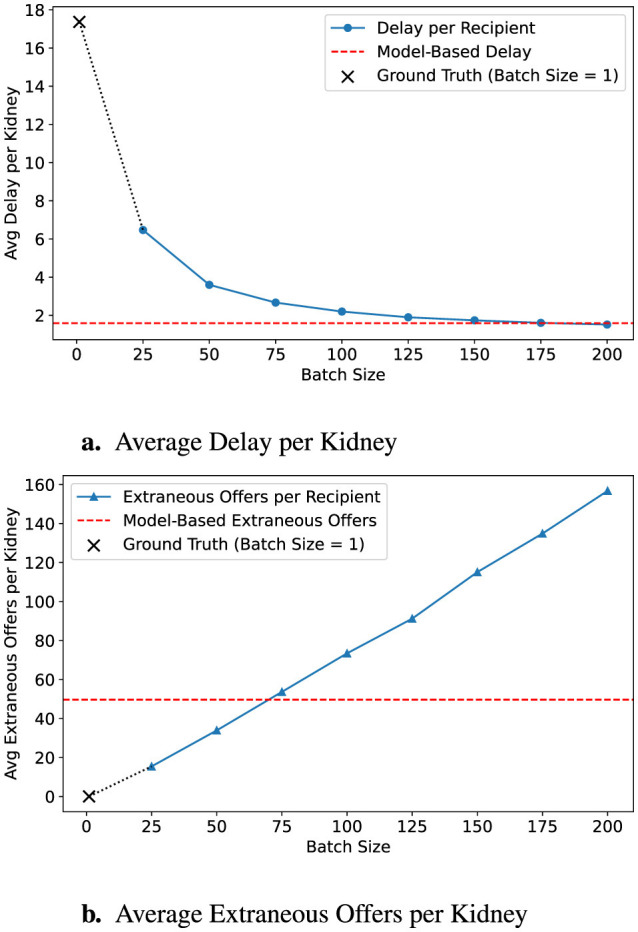
Impact of batch size on allocation efficiency. **(a)** Delay decreases with larger batches, but **(b)** extraneous offers increase. Ground-truth (*B* = 1) is shown as a **×**, and model predictions are in red dashed lines.

A key strength of our approach is its ability to generate organ-level predictions that inform real-time decisions in kidney allocation. To evaluate the practical impact of these predictions, we simulate allocation under a range of fixed batch sizes and compare outcomes against our dynamic batching model. As shown in Figure 2, smaller fixed batches limit the number of extraneous offers, potentially reducing decision fatigue, but result in substantial allocation delays. At the other extreme, large batch sizes significantly shorten placement times but generate excessive numbers of unnecessary offers, increasing cognitive burden on transplant teams. The current sequential match run system, which sends offers to one candidate at a time, minimizes extraneous offers entirely but leads to the longest delays. In contrast, our model offers a favorable trade-off between these extremes. By tailoring batch sizes based on predicted offer burden, it reduces the average time to allocation from 17.3 h to 1.59 h, while maintaining a low number of extraneous offers. This balance enables faster placement of organs without overwhelming clinicians, offering a scalable, data-driven alternative to one-size-fits-all batching strategies.

#### 3.2.1 OPO-configurable batch control

A critical contribution of our work is providing transplant professionals with flexible control over batching decisions, enabling practical adoption of our predictive framework. While our dynamic batching approach significantly improves organ placement times, and consequently the outcomes, clinical workflows often require adaptability to differing operational priorities. For instance, OPOs may seek to reduce simultaneous offer volume to mitigate decision fatigue and disappointment among surgeons and candidates. Alternatively, OPOs managing kidneys at high risk of nonuse might prioritize rapid allocation to minimize CIT.

To address these clinical needs, we propose two practical extensions: (i) a quantile-based survival threshold, allowing planners to shift predictions earlier or later in the match run, and (ii) a global batch-size multiplier, enabling direct adjustment of recommended batch sizes. These controls empower OPOs to tune batching aggressiveness according to institutional preferences, balancing allocation speed against operational and cognitive burden.

**Figure 3:**
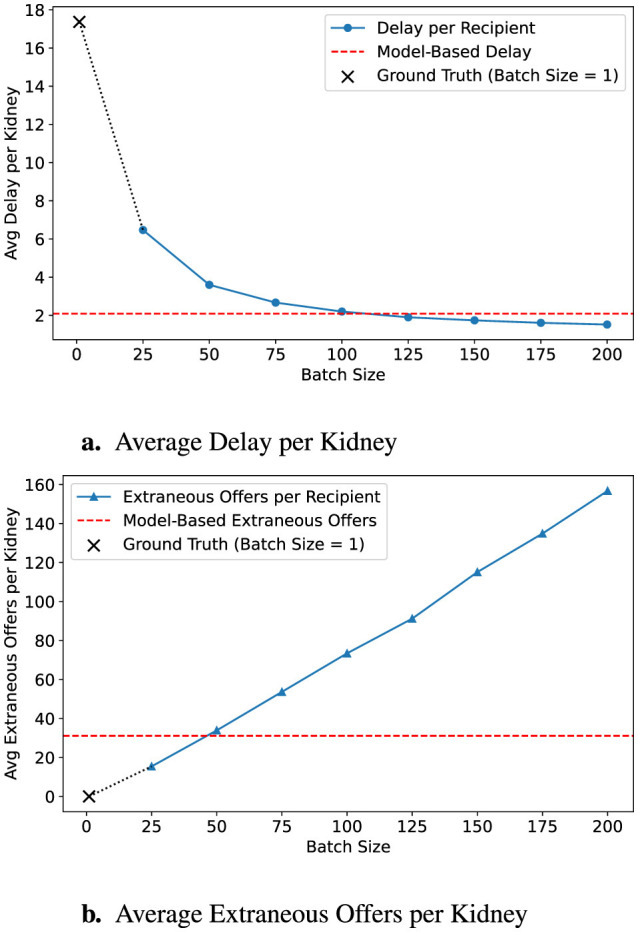
Predicted impact of early intervention policy. A survival threshold of 90% triggers earlier predictions, leading to smaller estimated batch sizes. **(a)** Delay is reduced, but **(b)** extraneous offers increase. Ground-truth is shown with a **×**; model targets with red dashed lines.

**Figure 4:**
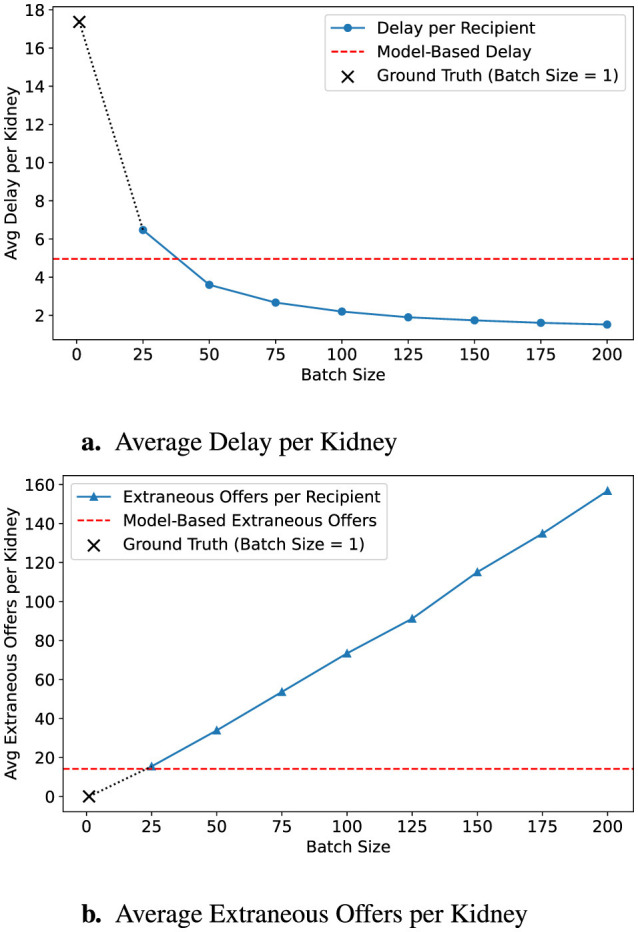
Predicted outcomes with conservative policy. This configuration uses an 80% survival threshold with a policy multiple of 0.5, scaling down batch size recommendations. **(a)** The resulting strategy delays fewer recipients, while **(b)** it also produces fewer extraneous offers. Ground-truth (*B* = 1) is marked with a **×**, and model-based targets are shown in red dashed lines.

Figure 3 demonstrates the effect of applying a conservative batching policy using a 90% survival threshold (α = 0.9). By shifting the predicted acceptance point earlier, the average allocation delay modestly increases to 2.09 h, while extraneous offers decrease substantially to 31.11. Figure 4 further illustrates a more conservative scenario combining α = 0.8 with a batch-size multiplier of 0.5, resulting in just 14.15 extraneous offers at the cost of 4.96 h average delay, outperforming even a fixed batch size of 25 across both dimensions.

These results underscore the model's ability to adapt dynamically to varied clinical objectives. OPOs can strategically choose more conservative configurations to limit decision fatigue and candidate disappointment or opt for aggressive settings to expedite placement when appropriate. By integrating such OPO- and organ-centric flexibility directly into the predictive framework, our approach serves as an effective decision-support tool tailored to real-world operational demands, ultimately facilitating smoother adoption in clinical practice.

### 3.3 Interpretability analysis

We conducted an interpretability analysis to better understand the contribution of individual features to model predictions and to assess whether a simpler, more transparent model could achieve comparable performance. As a first step, we evaluated a reduced feature set informed by clinical intuition and domain expertise. The motivation for this reduction is that simpler models, when carefully constructed, can improve interpretability, facilitate validation by clinicians, and increase trust in model outputs.

The reduced set includes mean values for several recipient and system-level characteristics: time on the waitlist, initial EPTS, distance from the donor hospital, and recipient age. To retain predictive strength, we also include mean CPRA and mean years on dialysis, along with their more extreme counterparts, namely, minimum CPRA and maximum years on dialysis, which capture additional heterogeneity in the recipient pool. Finally, key donor characteristics are represented by minimum Kidney Donor Risk Index (KDRI) and minimum CIT. This subset was selected to balance clinical relevance, interpretability, and predictive power.

Table 6 demonstrates that the reduced set retains strong predictive accuracy. Compared to the full model, the reduction yields only modest drops in time-dependent concordance (from 0.869 to 0.863) and *R*^2^ (from 0.923 to 0.915). This marginal decrease in performance greatly increases transparency and explainability.

**Table 6:** Comparison of RSF performance using all features vs. a reduced feature subset.

**Feature set**	**C-index**	**IBS**	**NBLL**	**MAE**	**MAPE**	**NRMSE**	**R^2^**	**RMSE**
**All features**	0.869	0.040	0.141	0.410	15.137	0.041	0.923	0.326
**Feature subset**	0.863	0.0319	0.105	0.405	15.536	0.041	0.915	0.329

**Figure 5:**
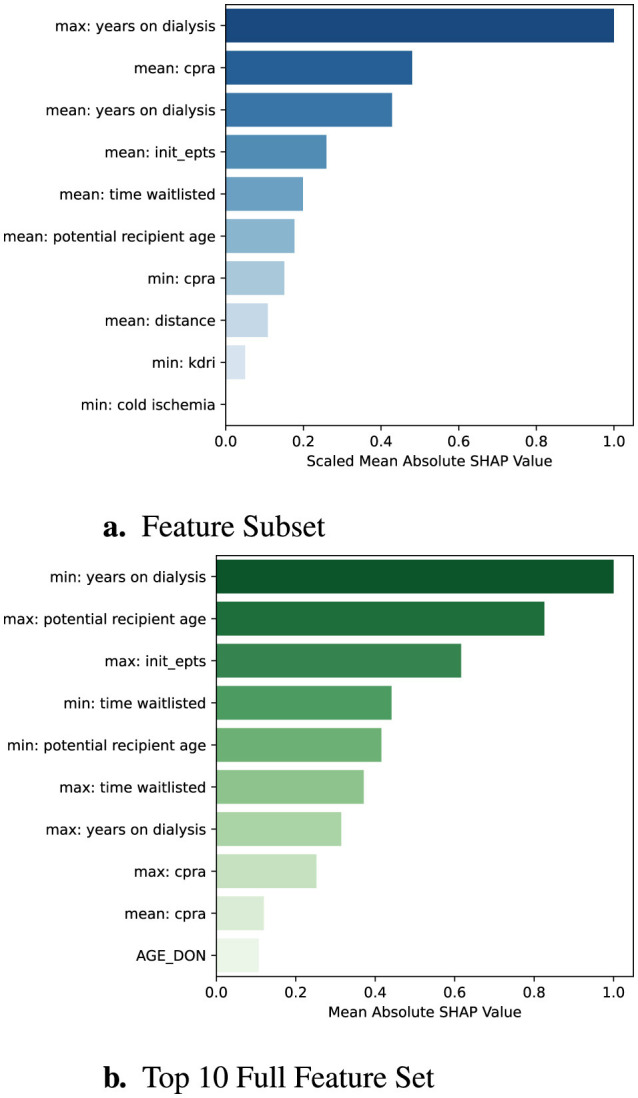
Global SHAP feature importance comparison. Each bar represents a feature's mean absolute SHAP value, scaled so that the most important feature is normalized to 1.0. **(a)** shows SHAP importances for the reduced feature set, while **(b)** shows the top 10 importances from the full feature set.

To understand the contribution of individual features to the predicted time until organ offer acceptance, we employed SHAP values. Figure 5 presents a global SHAP summary of the feature subset (left) as well as the top ten features in the full dataset ranked by their scaled mean absolute SHAP values. Notably, both the maximum and mean years on dialysis were the most influential predictors, reinforcing the clinical intuition that centers under pressure from long-waiting candidates are more likely to accept offers quickly. The strong overlap in the top-ranked features across both models indicates the reduced feature set retains many of the core drivers of model behavior. By emphasizing mean aggregations over extremes or percentiles, the reduced model may further enhance interpretability. Moreover, the model trained over feature subset performs comparably to the original model, supporting its use in settings where interpretability is deemed essential.

**Figure 6:**
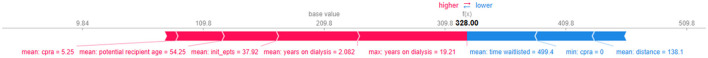
Local SHAP explanation for Example 1: a kidney with a high predicted offer burden of 328 offers. Strong upward pressure from maximum dialysis time and recipient frailty is partially offset by favorable pool-level features.

**Figure 7:**
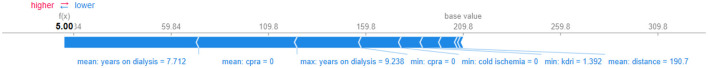
Local SHAP explanation for Example 2: a case with a low predicted offer burden of 5 offers. All key features contribute toward rapid acceptance.

**Figure 8:**
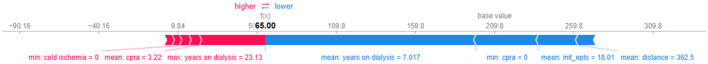
Local SHAP explanation for Example 3: a moderately difficult placement scenario with a predicted offer burden of 65. The model balances both positive and negative predictors to arrive at this estimate.

The interpretability gains from using a reduced feature set become particularly apparent when examining predictions for individual donor kidneys. To provide meaningful local explanations, we adapt the model's output so that SHAP values reflect the marginal contribution of each feature to the predicted number of offers required before acceptance. While the original model produces a risk score where higher values imply shorter time-to-event, this representation is less intuitive for SHAP-based interpretation. Instead, we apply a filtering function to output the predicted median number of offers, making the explanations more actionable and interpretable in the context of organ allocation. Figures 6, 7, and 8 present local SHAP explanations for three kidneys with varying predicted offer burdens. Each plot visualizes the influence of individual features, showing how they either increase or decrease the predicted number of offers relative to a dataset-specific baseline.

#### 3.3.1 Example 1: high predicted offer burden (328 offers)

Figure 6 illustrates a case with a high predicted offer burden. Several features contribute significantly to increasing the number of expected offers before acceptance. Most notably, this includes the maximum years on dialysis, highlighting a candidate who began dialysis over 19 years ago, paired with a mean dialysis duration of 2.08 years across the pool. An elevated mean EPTS score (37.92) and recipient age (54.25) further reinforce the model's prediction of slow placement. Conversely, favorable allocation indicators such as a low mean time on the waitlist (499.4 days), minimal CPRA, and short donor-recipient distance serve to reduce the predicted value. This case reflects a complex trade-off between competing clinical signals.

#### 3.3.2 Example 2: low predicted offer burden (5 offers)

Figure 7 presents a highly favorable allocation scenario. All dominant features exert downward pressure on the prediction, resulting in a very low expected number of offers. The model attributes this to a combination of highly favorable recipient and donor characteristics, including a mean CPRA of 0, an elevated mean dialysis duration of 7.71 years, and a low KDRI of 1.39. Additionally, a CIT of 0 suggests that offers were initiated promptly, enhancing the chances of early acceptance. This case highlights a scenario where both medical urgency and organ quality align to produce fast placement.

#### 3.3.3 Example 3: moderate predicted offer burden (65 offers)

Figure 8 presents a more balanced case with a moderate predicted offer burden. The strongest positive influence comes from a maximum dialysis duration of 23.13 years—suggesting at least one difficult-to-place candidate in the offer pool. However, this is partially counteracted by several favorable features, including a low minimum CPRA, a low initial EPTS of 18.01, and an elevated mean dialysis duration of 7.02 years. This case demonstrates how the model integrates both conflicting and reinforcing signals to produce a nuanced, context-sensitive estimate.

## 4 Discussion and conclusion

In the current kidney allocation system, offers are typically made sequentially. While this limits decision fatigue by stopping the process once an organ is accepted, it often results in substantial delays and contributes to high rates of organ nonuse. To mitigate these delays, simultaneously expiring offers have been proposed as an alternative. However, when implemented with a fixed batch size, this approach can overwhelm transplant teams with excessive evaluations, leading to decision fatigue and diminished effectiveness. To balance the strengths of both strategies, we developed a machine learning framework that predicts the number of offers a deceased donor kidney will likely require before acceptance. These individualized predictions enable dynamic batch sizing tailored to each organ. Using real-world match run data, we demonstrated strong predictive accuracy and evaluated the operational impact of our method through simulations. The results show that dynamically adjusted batch sizes can substantially reduce placement delays while controlling the number of unnecessary offers. To support clinical transparency and trust, we employed SHAP values to interpret the model's predictions and highlight the most influential features driving offer burden.

Previous studies on batching strategies in kidney allocation typically applied static batch sizing or relied on large-scale simulations to optimize batches based on predefined organ categories or locations (Erazo et al., 2022; Mankowski et al., 2019). In contrast, our work introduces the novel use of survival-based machine learning algorithms to generate real-time, individualized predictions at the organ level. This dynamic approach enables fine-tuned allocation strategies, balancing efficiency and clinical workload to reduce organ nonuse rates without overwhelming transplant professionals. Our simulation results demonstrate clear advantages over static batching policies. The proposed dynamic batching method reduced average offer delay from 17.37 h (under sequential allocation) to just 1.59 h, achieving outcomes comparable to very large batch sizes but with significantly fewer extraneous offers, similar to smaller batches.

The developed framework serves as a clinical decision-support tool, helping transplant professionals navigate the complexities introduced by circle-based allocation (Adler et al., 2021; Jay and Stratta, 2023). Configurable parameters such as model aggressiveness and batch-size multipliers allow clinicians to adjust predictions according to operational priorities. Additionally, the incorporation of local and global SHAP interpretations enhances clinical trust by providing transparent explanations that align predictions with clinical intuition (Nasarian et al., 2024). Real-world deployment would require operational and policy alignment. The model would need to be integrated into existing allocation systems ideally with the capability to update predictions in real time as match runs progress. Implementation would further require coordination with OPOs, transplant centers, and regulatory bodies to ensure compliance with allocation policy and avoid unintended inequities in access.

Despite promising outcomes, several limitations exist for our work. First, our model's performance is derived from retrospective registry data; thus, real-world validation through prospective studies or controlled pilots is necessary to confirm practical feasibility and broader system-level benefits. Second, because we aggregate offers at the donor level, CIT is treated statically at the initial offer stage rather than as a dynamically accumulating constraint. Future models should explicitly account for evolving CIT and its impact on downstream acceptance probabilities. Third, our engineered features summarize characteristics of candidate pools based on actual offer history, potentially biasing predictions since candidate composition may change depending on acceptance timing. Since we use the offer history, candidate pool features do not account for potential changes in waitlist composition over time. Conversely, this approach captures actual operational constraints and decision processes that are often absent from studies relying entirely on simulated candidate lists. Relatedly, reliance on historical allocation and acceptance data may embed biases from prior allocation practices and center decision-making behaviors. While this grounding in observed behavior reflects real-world operational constraints, it also risks perpetuating inequities present in historical patterns. Future extensions could mitigate this limitation by incorporating explicit center-level modeling or by testing the framework prospectively. Furthermore, our model implicitly incorporates historical center decision-making behaviors without explicitly modeling these patterns or adapting dynamically to shifts in behavior over time. Exploring novel models that allow for incorporating explicit center identifiers is left for future research. Finally, we note that the model is trained exclusively on U.S. kidney allocation data. Adapting to other operational infrastructures would likely require model retraining and validation in those contexts.

In future research, we aim to validate this framework through prospective simulation studies or pilot implementation to provide robust evidence of clinical feasibility and impact. Additionally, extending the current model to incorporate iterative, auto-regressive predictions within individual match runs could further enhance real-world applicability. Specifically, integrating prior batch outcomes, accumulated CIT, and evolving candidate pool characteristics into subsequent predictions could improve adaptive decision-making. Lastly, exploring integration strategies into existing clinical decision-support systems represents a critical step toward practical implementation in transplant workflows.

## Data Availability

Publicly available datasets were analyzed in this study. This data can be found at: we utilize a combined dataset composed of the deceased donor data and the Potential Transplant Recipient (PTR) dataset from Organ Procurement and Transplantation Network (OPTN) which documents all kidney offers to patients on the U.S. waiting list. The datasets can be requested from the OPTN. https://optn.transplant.hrsa.gov/data/.
